# Prostate Cancer Treatment in the Extreme Apex: Preservation of Continence With Robotic-Assisted MRI-Guided Transurethral Ultrasound Ablation

**DOI:** 10.7759/cureus.108671

**Published:** 2026-05-11

**Authors:** Victoria Bird, Kathy Busch, Roland Rose, Juan Varela, Joseph Busch

**Affiliations:** 1 Urology, National Medical Association and Research Group, Gainesville, USA; 2 Urology, HCA Florida North Florida Hospital, Gainesville, USA; 3 Interventional Radiology, Busch Center, Alpharetta, USA; 4 Urology, University of Florida College of Medicine, Gainesville, USA

**Keywords:** cancer in deep apical prostate, cancer involving sphincter of apical prostate, preservation of continence, prostate apex, prostate cancer, prostate cancer treatment, successful oncologic treatment of apical prostate cancer

## Abstract

Purpose

MRI-guided transurethral ultrasound ablation (TULSA) is a novel, minimally invasive therapy for prostate cancer designed to preserve urinary continence and erectile function. The original TULSA pivotal trial mandated preservation of 3 mm of apical tissue to protect the external sphincter. We evaluated oncologic and functional outcomes in patients with prostate cancer located at the extreme apex, including lesions abutting or involving the sphincter.

Materials and methods

We performed a retrospective analysis of a subgroup from a prospective TULSA cohort at a single center. Patients included had MRI-visible extreme apical lesions abutting or involving the external sphincter and ≥6 months of follow-up with prostate-specific antigen (PSA) or MRI. The extreme apex is considered to be the most distal, tapering portion of the gland adjacent to the prostatic urethral termination and membranous urethra, immediately proximal to the external urinary sphincter (rhabdosphincter).

Treatment planning incorporated intraoperative diffusion-weighted imaging (DWI), apparent diffusion coefficient (ADC) maps, and T2-weighted imaging. For lesions near the sphincter, a reduced 5 mm safety margin was applied, treating ≤50% of sphincter length or arc. Follow-up included serial PSA testing every three months and MRI, International Prostate Symptom Score (IPSS), and International Index of Erectile Function (IIEF) at six to nine months. Local recurrence was assessed using Prostate Imaging for Recurrence Reporting (PI-RR) criteria.

Results

Sixty-eight patients (61 primary and 7 salvage) were treated. Median age was 63 years, with a median follow-up of 12 months. PSA declined from a median of 7 ng/mL to 0.5 ng/mL. Among 59 patients with follow-up MRI, 88% demonstrated no evidence of disease. Six of seven patients with suspicious imaging underwent repeat TULSA, with favorable early outcomes. Functional outcomes were excellent: all patients were pad-free by three months, 80% maintained erections sufficient for penetration, and IPSS scores remained stable. Adverse events were mild and self-limited; no rectal injuries occurred.

Conclusions

MRI-guided TULSA is a safe and effective treatment for prostate cancer involving the extreme apical region. Despite the technical challenges posed by tumors abutting or partially involving the external urinary sphincter, carefully planned and controlled ablation - limited to ≤50% of sphincter involvement - can achieve excellent oncologic outcomes, with no evidence of residual disease on follow-up imaging and no suggestion of positive margins in treated patients. Importantly, this approach preserves urinary continence, with all patients remaining pad-free, while maintaining favorable functional outcomes. These findings support the feasibility of TULSA for the treatment of even bulky apical tumors without compromising cancer control or quality of life.

## Introduction

Prostate cancer remains one of the most commonly diagnosed malignancies among men worldwide, with treatment decisions often balancing oncologic control against preservation of urinary and sexual function [1]. Radical prostatectomy and radiation therapy are associated with substantial risks of urinary incontinence and erectile dysfunction, particularly when tumors are located near the prostatic apex [2-4].

The apical region poses a unique therapeutic challenge due to its close anatomical relationship with the external urethral sphincter [5]. Preservation of sphincter integrity is critical for maintaining continence, yet undertreatment risks residual disease [6]. Consequently, conventional therapies often compromise between oncologic efficacy and functional outcomes.

Robotic-assisted MRI-guided transurethral ultrasound ablation of the prostate (TULSA-PRO) has emerged as a promising minimally invasive modality that allows real-time thermometry and precise tissue ablation [7-9]. The TACT study (pivotal study of TULSA) demonstrated effective whole-gland ablation with low morbidity; however, the protocol required sparing a 3 mm apical margin to protect the sphincter, potentially limiting treatment of apical tumors [10].

Recent advances in multiparametric MRI (mpMRI) and real-time intraoperative imaging enable improved visualization of both tumor and sphincter/urethral anatomy [11,12]. These developments raise the possibility of safely treating tumors at the extreme apex while preserving continence.

In this study, we report oncologic, imaging, and functional outcomes in a cohort of patients undergoing TULSA for prostate cancer lesions located at the extreme apex, including tumors abutting or involving the external sphincter.

## Materials and methods

We performed a retrospective analysis of a prospectively maintained, single-center cohort of patients who underwent MRI-guided TULSA for prostate cancer. The objective of this study was to evaluate oncologic and functional outcomes in men with MRI-visible prostate cancer located at the extreme apex, including lesions abutting or involving the external urethral sphincter. The extreme apex is defined as the most distal, tapering portion of the gland adjacent to the prostatic urethral termination and membranous urethra, immediately proximal to the external urinary sphincter (rhabdosphincter). It represents the inferior-most extent of prostatic tissue, where the gland narrows and interfaces with the urogenital diaphragm.

Eligible patients included men with MRI-visible apical prostate cancer treated with robotic-assisted MRI-guided TULSA who had at least six months of follow-up with prostate-specific antigen (PSA) testing and/or mpMRI. Both primary and salvage treatment cases were included.

**Figure 1:**
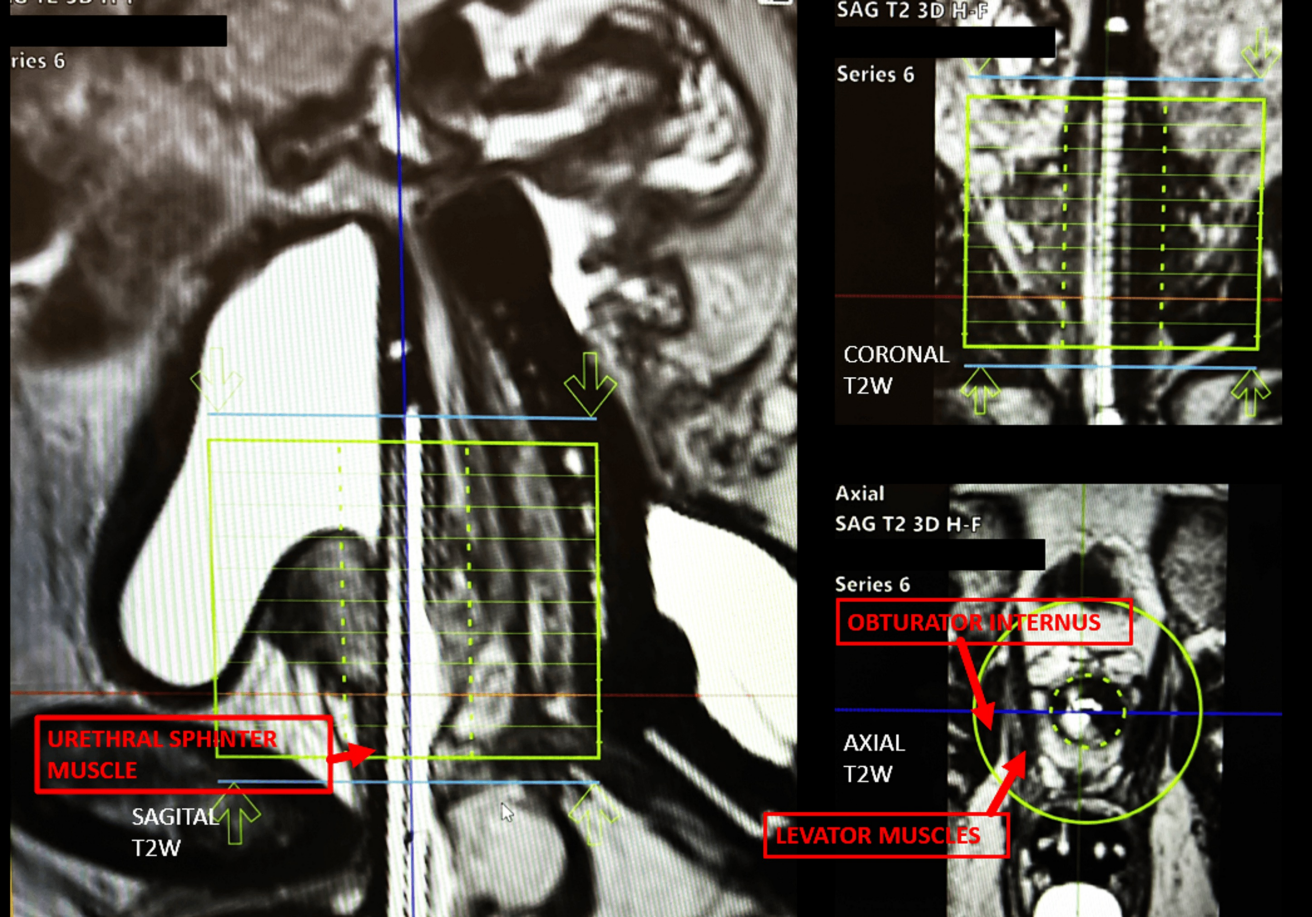
TULSA-PRO software and prostatic anatomic landmarks Representative axial, coronal, and sagittal planning images from the transurethral ultrasound ablation of the prostate (TULSA-PRO) platform demonstrating the urethral applicator, rectal cooling device, and key anatomic landmarks relevant to treatment of apical prostate tumors, including the levator ani and obturator internus muscles. Accurate identification of these structures is critical for treatment planning and preservation of continence.

**Figure 2:**
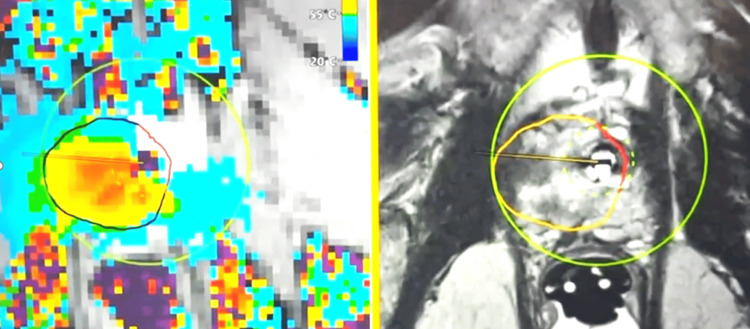
Initial treatment planning and "First-pass" ablation of an apical prostatic tumor T2-weighted image demonstrating a right apical prostatic tumor during real-time transurethral ultrasound ablation (TULSA) treatment. Colorimetric MR thermometry demonstrates ablative temperatures of 55°C-86°C within the planned treatment margin (orange line).

Treatment planning incorporated mpMRI, including T2-weighted imaging, diffusion-weighted imaging (DWI), and apparent diffusion coefficient (ADC) maps, to delineate tumor boundaries and define sphincter anatomy. For lesions adjacent to the external urethral sphincter, a reduced treatment margin of 5 mm was used, with ablation limited to ≤50% of the sphincter length or circumferential arc in an effort to preserve continence. Figure 1 illustrates relevant prostatic anatomic landmarks on TULSA planning software, and Figure 2 demonstrates tumor localization, segmentation, and initial treatment planning.

**Figure 3:**
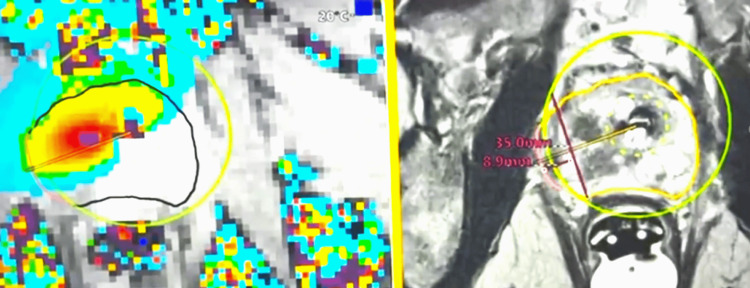
"Second pass" treatment with re-segmentation of the prostate Post-initial treatment image demonstrating tissue swelling and re-segmentation before a “second treatment” pass. The updated treatment plan incorporates an additional 8.9 mm margin from the tumor edge (red line), with real-time thermometry confirming treatment coverage of the target and planned margin.

Real-time MR thermometry was used intraoperatively to monitor thermal delivery. After the initial treatment pass, treatment geometry was reassessed and, when indicated, a second pass was performed with re-segmentation to optimize energy delivery, as tissue swelling and treatment-related shift of the urethral applicator could alter alignment (Figure 3).

Post-treatment follow-up included clinical monitoring during the first two weeks, PSA measurement every three months, and mpMRI, International Prostate Symptom Score (IPSS), and International Index of Erectile Function (IIEF) assessment at six to nine months. Local recurrence was assessed using Prostate Imaging for Recurrence Reporting (PI-RR) criteria.

Continuous variables are reported as mean ± standard deviation (SD), median with interquartile range (IQR), and range, as appropriate. Categorical variables are reported as frequency and percentage. Missing data were recorded for variables with incomplete follow-up. All statistical analyses were performed using JASP Software (version 0.19.3; JASP Team, Amsterdam, the Netherlands). Paired pre- and post-treatment comparisons were performed using the Wilcoxon signed-rank test, because several variables demonstrated non-normal distributions on Shapiro-Wilk testing.

This retrospective analysis of de-identified clinical data was reviewed by the institutional review board and determined to be exempt.

## Results

A total of 68 patients were included in the study cohort, comprising 61 primary treatment cases (89.7%) and seven salvage treatment cases (10.3%), including prior focal laser ablation (n = 5), high-intensity focused ultrasound (n = 1), and radiation therapy (n = 1). Baseline clinical characteristics are summarized in Table 1. Paired pre- and post-treatment analyses demonstrated significant reductions in prostate volume and PSA following treatment. Median prostate volume decreased from 42.5 cc to 15 cc (Wilcoxon signed-rank, p < 0.001). Baseline PSA declined significantly to both the last follow-up PSA and nadir PSA values (both p < 0.001). No significant change in urinary symptom burden was observed between baseline and follow-up IPSS scores (p = 0.582). Post-treatment erectile function scores remained favorable, with recovery to high post-treatment IIEF-Q2 values in evaluable patients (p < 0.001).

**Table 1:** Baseline demographic, oncologic, imaging, and functional characteristics of the study cohort Continuous variables are reported as mean ± standard deviation (SD), median (interquartile range (IQR)), and range. Categorical variables are reported as frequency and percentage. * Available in 64 patients; † Available in 60 patients; ‡ Available in 67 patients; § Available in 46 patients. IPSS: International Prostate Symptom Score; IIEF: International Index of Erectile Function; FLA: Focal Laser Ablation; HIFU: High-Intensity Focused Ultrasound; RT: Radiation Therapy

Variable	N	Mean ± SD	Median (IQR)	Range
Age, years	68	62.9 ± 8.7	62.5 (10.0)	31-85
Baseline Grade Group*	64	2.61 ± 0.97	2 (1)	1-5
PI-RADS score†	60	4.70 ± 0.46	5 (1)	4-5
Prostate volume, cc	68	48.6 ± 25.8	42.5 (23.0)	12-129
Baseline PSA, ng/mL	68	10.0 ± 15.2	7.15 (6.03)	1.7-127
Baseline IPSS‡	67	8.51 ± 6.94	7 (9)	0-32
Baseline IIEF-Q2§	46	3.63 ± 1.64	4 (2)	0-5
Treatment Indication				N (%)
Primary treatment				61 (89.7)
Salvage treatment				7 (10.3)
FLA salvage				5 (7.4)
HIFU salvage				1 (1.5)
RT salvage				1 (1.5)

Median age was 62.5 years (IQR 10.0), and mean age was 62.9 ± 8.7 years. Median baseline prostate volume was 42.5 cc (IQR 23.0), with a mean volume of 48.6 ± 25.8 cc. Median baseline PSA was 7.15 ng/mL (IQR 6.03), while mean baseline PSA was 10.0 ± 15.2 ng/mL. Among patients with available data, the median baseline Grade Group was 2 (IQR 1), and the median PI-RADS score was 5 (IQR 1). Baseline urinary and sexual function were favorable overall, with a median IPSS of 7 (IQR 9) and a median IIEF-Q2 score of 4 (IQR 2).

Post-treatment clinical outcomes are summarized in Table 2. Median follow-up was 12.0 months (IQR 8.37), with a mean follow-up of 13.7 ± 7.25 months. Median last PSA was 0.90 ng/mL (IQR 1.83), declining from a baseline median of 7.15 ng/mL. Median PSA nadir was 0.50 ng/mL (IQR 0.73). Median follow-up prostate volume was 15 cc (IQR 12), corresponding to a median prostate volume reduction of 60.4% (IQR 19.9).

**Table 2:** Post-treatment clinical outcomes following MRI-guided TULSA Post-treatment oncologic, volumetric, urinary, and sexual function outcomes following MRI-guided transurethral ultrasound ablation (TULSA). Continuous variables are reported as mean ± standard deviation (SD), median (interquartile range (IQR)), and range. Categorical variables are reported as frequency and percentage. * Available in 49 patients; † Available in 48 patients; ‡ Available in 48 patients; § Available in 41 patients. IPSS: International Prostate Symptom Score; IIEF: International Index of Erectile Function; PSA: Prostate-Specific Antigen

Variable	N	Mean ± SD	Median (IQR)	Range
Last PSA, ng/mL	67	2.09 ± 3.75	0.90 (1.83)	0-21.5
PSA nadir, ng/mL	68	0.85 ± 1.21	0.50 (0.73)	0-7.6
Follow-up prostate volume, cc *	49	19.3 ± 13.0	15 (12)	6-78
Prostate volume reduction, % †	48	58.6 ± 16.4	60.4 (19.9)	8.6-89.3
Total follow-up, months	67	13.7 ± 7.25	12.0 (8.37)	5.1-45.4
Last IPSS ‡	48	8.31 ± 6.17	6 (8)	1-28
Highest post-treatment IIEF-Q2 §	41	3.34 ± 1.48	4 (1)	0-5

Functional outcomes remained favorable following treatment. Median last IPSS was 6 (IQR 8), suggesting stable lower urinary tract symptoms during follow-up. Median highest post-treatment IIEF-Q2 score was 4 (IQR 1), indicating preservation of erectile function in many patients. All patients achieved pad-free continence by three months. Erectile function sufficient for penetration was preserved in 80% of evaluable patients.

Among 59 patients with follow-up MRI available, 52 (88.1%) demonstrated no radiographic evidence of disease. Seven patients demonstrated suspicious imaging findings (PI-RR 4-5). Of these, six underwent repeat TULSA, with five demonstrating favorable early post-retreatment outcomes and one remaining under active surveillance.

**Figure 4:**
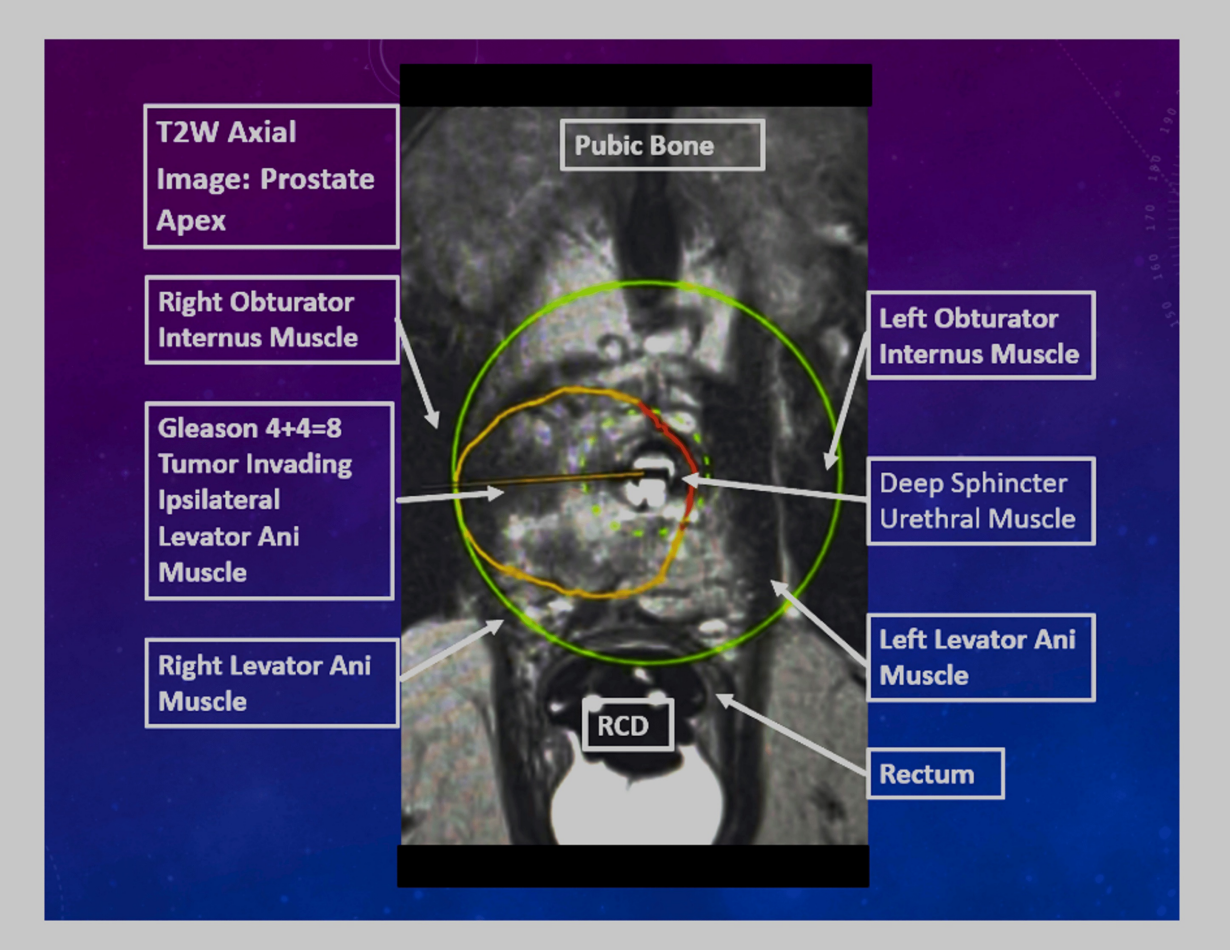
Right-sided Gleason 4 + 4 = 8 apical tumor involving the ipsilateral levator ani muscle Representative axial T2-weighted planning image demonstrating a right apical prostate tumor with extension into the ipsilateral levator ani muscle. The green circle represents the available treatment field. The orange contour indicates the planned treatment zone. The red contour denotes a no-treatment zone designed to preserve the striated urethral sphincter, contralateral levator ani, and obturator internus musculature. RCD: Rectal Cooling Device

Figure 4 demonstrates a representative right-sided Gleason 4 + 4 = 8 apical tumor with extension into the ipsilateral levator ani muscle. Treatment planning illustrates selective targeting of the lesion while preserving the striated sphincter complex, contralateral levator ani, and obturator internus musculature through designated no-treatment zones.

**Figure 5:**
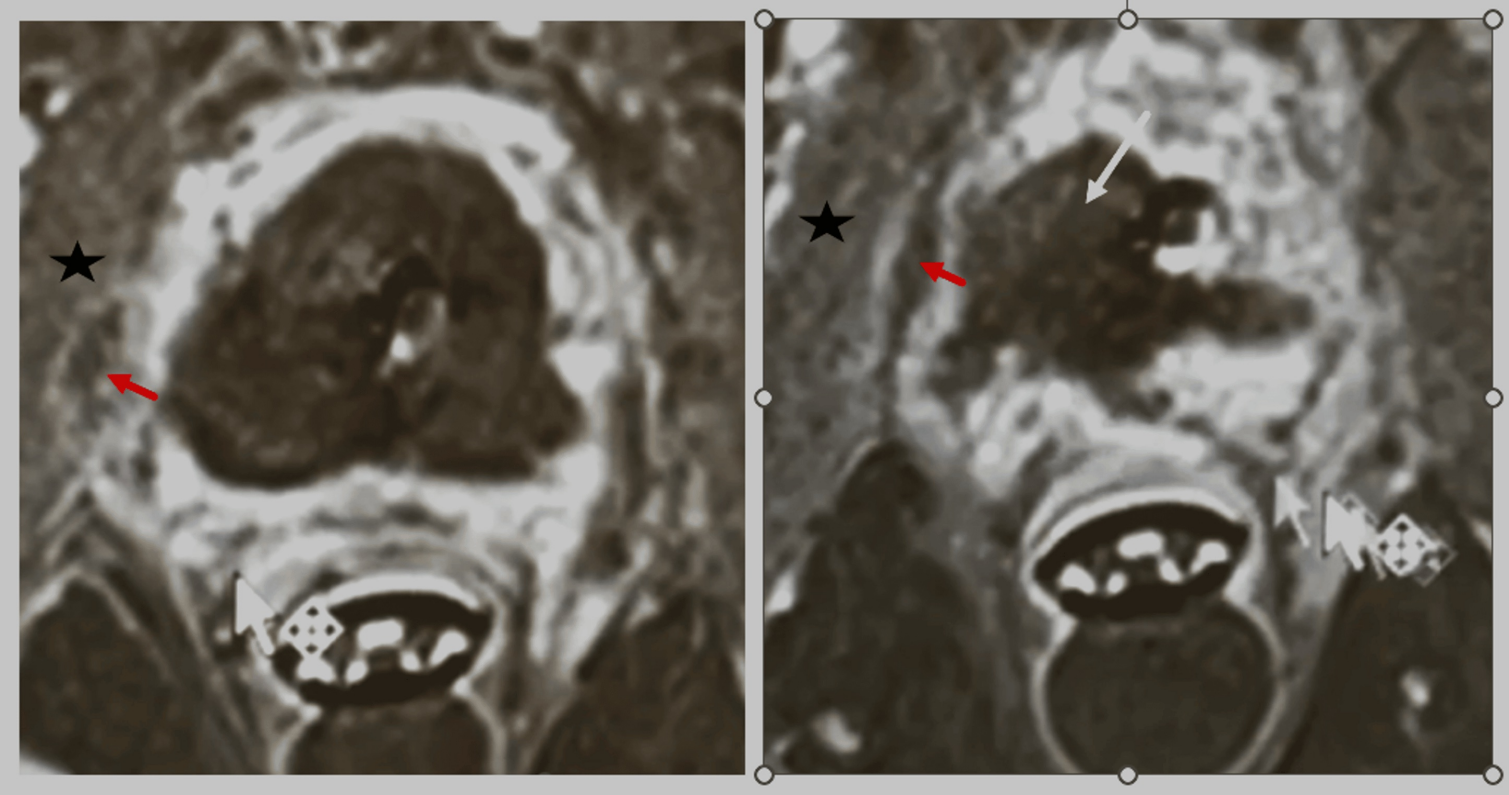
Post-ablation contrast-enhanced axial T1-weighted MRI Post-treatment contrast-enhanced axial T1-weighted images following transurethral ultrasound ablation (TULSA). The left panel demonstrates the expected treatment effect involving the obturator internus muscle superior to the ablation zone (star; red arrow). The right panel demonstrates the absence of contrast enhancement within the treated apical lesion (white arrow), consistent with successful ablation, with adjacent treatment effect involving the ipsilateral levator ani and obturator internus musculature (star; red arrow).

Figure 5 demonstrates post-ablation contrast-enhanced axial T1-weighted imaging. The treated lesion shows absence of contrast enhancement, consistent with successful focal ablation, with the expected adjacent treatment effect involving the ipsilateral levator ani and obturator internus musculature.

Adverse events were generally mild and self-limited, including transient lower urinary tract symptoms (2.9%), hematuria (5.9%), and bladder spasms (1.5%), resolving within four weeks. Three patients developed urinary retention or obstruction requiring endoscopic intervention within five months of treatment. No rectal injuries, fistulae, or treatment-related complications were observed.

## Discussion

Management of prostate cancer at the extreme apex remains a significant clinical challenge due to the close anatomical relationship between the tumor and the external urethral sphincter. Preservation of continence is critically dependent on maintaining sphincter integrity, yet traditional therapies often require aggressive apical dissection or high-dose radiation in this region, increasing the risk of functional compromise [2,3]. As a result, clinicians are frequently faced with a difficult balance between achieving adequate oncologic control and minimizing treatment-related morbidity. This challenge is particularly pronounced in patients with tumors that directly abut or involve the sphincter, where even small deviations in treatment margins can have meaningful functional consequences.

Our findings suggest that TULSA provides a compelling solution to this clinical dilemma by enabling precise and controlled ablation of apical tumors while preserving sphincter function. Through the use of real-time MRI guidance and a deliberate strategy of limiting ablation to ≤50% of the sphincter, we were able to achieve excellent functional outcomes, with all patients remaining pad-free by three months. This degree of continence preservation compares favorably with outcomes reported following radical prostatectomy, where incontinence rates range from 5% to 20% depending on patient factors and surgical technique [13,14]. These results highlight the potential of TULSA to mitigate one of the most impactful adverse effects associated with conventional therapies, particularly in anatomically high-risk regions such as the apex.

**Figure 6:**
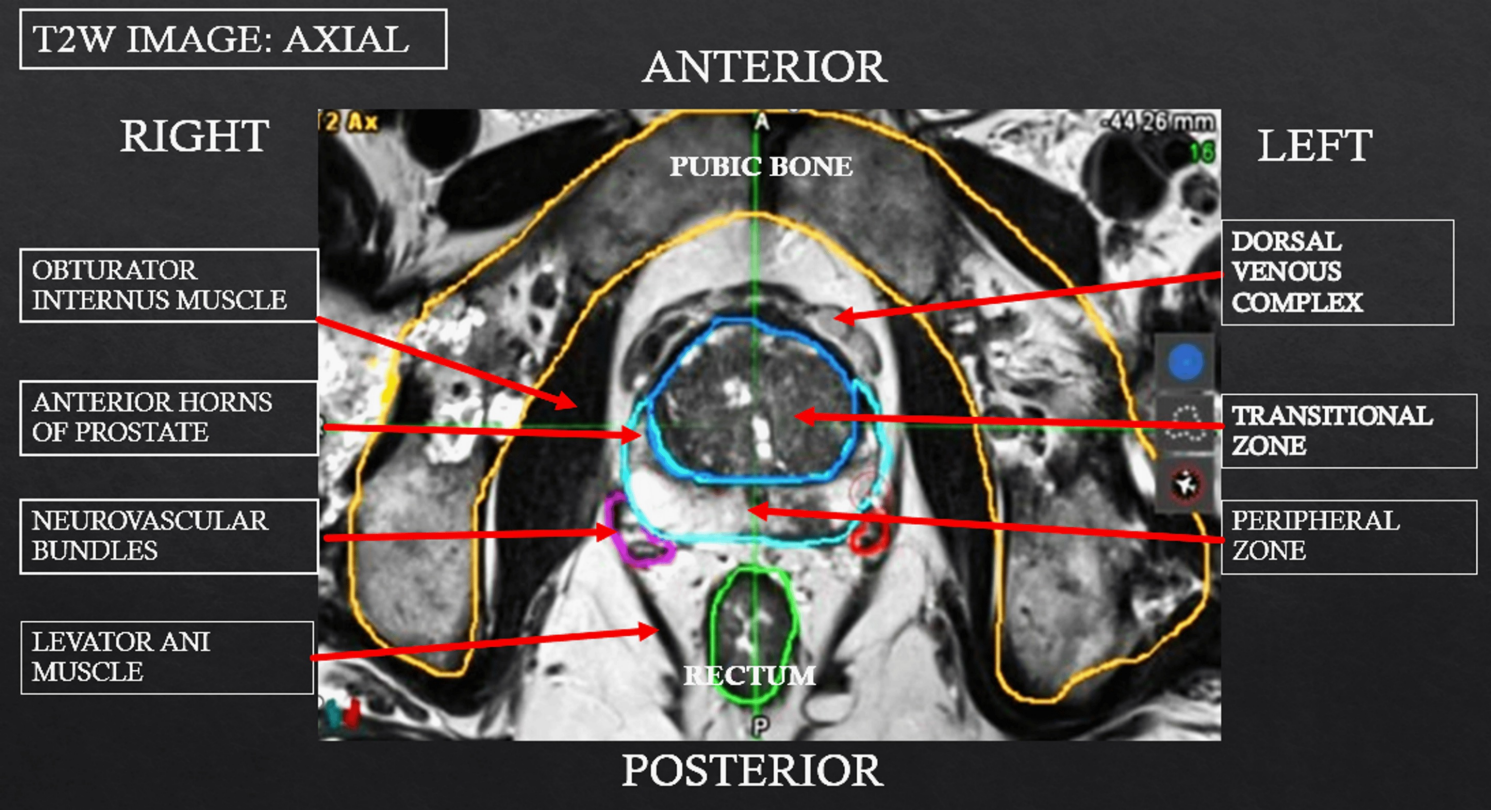
Advances in MRI software segmentation allows identification of key prostatic landmarks In this figure, an axial image depicts key structures related to primary and secondary continence control, including the levator ani muscles and obturator internus muscles.

The oncologic outcomes observed in this cohort further support the utility of this approach. The high rate of negative follow-up MRI (88%) is consistent with results from the TULSA pivotal trial and aligns with outcomes reported for other focal therapy modalities [10,15]. Notably, the ability to perform repeat TULSA in patients with early suspicious imaging findings underscores an important advantage of focal therapy strategies. Rather than precluding future treatment options, TULSA allows for iterative management while preserving the underlying organ and minimizing cumulative toxicity. This flexibility may be especially valuable in patients with localized recurrences or multifocal disease patterns. The integration of mpMRI into both treatment planning and follow-up was central to the success of this approach. Advanced imaging modalities, including DWI and ADC mapping, enable high-resolution visualization of tumor extent and adjacent critical structures. This facilitates accurate targeting of apical cancers while maintaining safe margins around the sphincter, even in complex anatomical scenarios [11,16]. Figure 6 represents the segmentation of different sections of the prostate in order to facilitate the identification of prostatic structures (MIMS V 7.3.4; MIM Software Inc., Cleveland, OH, USA). The ability to dynamically adapt treatment plans based on intraoperative imaging further enhances precision and may contribute to the favorable balance between oncologic control and functional preservation observed in this study.

Functional preservation remains a key advantage of TULSA and a major driver of its clinical appeal. In addition to excellent continence outcomes, erectile function was maintained in 80% of patients, which is consistent with prior reports demonstrating superior sexual outcomes with focal therapies compared to surgery and radiation [17-20]. Given the significant impact of sexual dysfunction on quality of life, these findings reinforce the value of minimally invasive, image-guided approaches that prioritize both cancer control and patient-centered outcomes.

This study addresses a clinically challenging and high-risk anatomical region - the prostatic apex - where treatment precision is critical. The use of real-time MRI thermometry and clearly defined adaptive treatment planning enhances procedural accuracy and reproducibility. Additionally, the study demonstrates excellent functional outcomes, particularly with respect to continence preservation, and highlights the feasibility of repeat TULSA in cases requiring re-intervention.

This study has several limitations that warrant consideration. The retrospective design introduces the potential for selection bias, and the single-center experience may limit generalizability to broader patient populations and practice settings. The findings should also be interpreted in the context of a relatively short follow-up period. The absence of systematic post-treatment biopsy limits definitive assessment of oncologic control. The lack of uniform biopsy confirmation of MRI findings is another limitation, as imaging alone may not fully capture residual or recurrent disease in all cases. Additionally, the small size of the salvage subgroup restricts the generalizability of conclusions in this population. The relatively short follow-up period restricts assessment of long-term oncologic durability, which remains a critical endpoint in prostate cancer management.

Despite these limitations, the results of this study provide important preliminary evidence supporting the feasibility and effectiveness of TULSA in treating prostate cancer at the extreme apex. The combination of precise tumor ablation, excellent continence outcomes, and preservation of erectile function suggests that this approach may help address a longstanding gap in the management of apical disease [21,22]. Future prospective, multi-institutional studies with longer follow-up are needed to validate these findings, optimize patient selection, and further refine treatment parameters [23], including safety margins and retreatment strategies.

## Conclusions

MRI-guided TULSA is a safe and effective treatment for prostate cancer located at the extreme apex. A controlled ablation strategy involving ≤50% of the external sphincter muscles preserves urinary continence while maintaining favorable oncologic outcomes. The findings from this study suggest that MRI-guided TULSA is a highly promising treatment option for patients with prostate cancer located at the extreme apex, even in cases where the tumor abuts or involves the external sphincter. Despite the technical challenges associated with treating this anatomically sensitive region, the approach demonstrated strong early oncologic control, with a substantial proportion of patients showing no evidence of disease on follow-up imaging and marked reductions in PSA levels. The ability to safely deliver targeted ablation in such close proximity to the sphincter while maintaining effective cancer control highlights the precision and adaptability of this technique.

Equally important, the functional outcomes observed in this cohort underscore the potential of TULSA to preserve quality of life. All patients achieved pad-free continence within three months, and a large majority maintained erectile function sufficient for penetration, with stable urinary symptom scores over time. These results are particularly encouraging given the historical trade-offs between oncologic efficacy and functional preservation in prostate cancer therapies. Together, the data support TULSA as a minimally invasive and patient-centered treatment strategy that not only offers promising cancer control but also successfully spares urinary continence and sexual function, warranting further investigation in larger and longer-term studies. This approach may represent a paradigm shift in the management of apical prostate cancer.
